# Indacaterol improves lung hyperinflation and physical activity in patients with moderate chronic obstructive pulmonary disease - a randomized, multicenter, double-blind, placebo-controlled study

**DOI:** 10.1186/1471-2466-14-158

**Published:** 2014-10-04

**Authors:** Henrik Watz, Felix Krippner, Anne Kirsten, Helgo Magnussen, Claus Vogelmeier

**Affiliations:** Pulmonary Research Institute at LungClinic Grosshansdorf, Airway Research Center North, Member of the German Center for Lung Research, Woehrendamm 80, D-22927 Grosshansdorf, Germany; Novartis Pharma GmbH, Nuremberg, Germany; Department of Respiratory Medicine, University of Marburg, University Giessen and Marburg Lung Center, Member of the German Center for Lung Research, Marburg, Germany

**Keywords:** Chronic obstructive pulmonary disease, Lung hyperinflation, Bronchodilator treatment, Physical activity

## Abstract

**Background:**

Indacaterol is a long-acting beta-2 agonist for once-daily treatment of COPD. We evaluated the effects of indacaterol 150 μg on lung hyperinflation compared with placebo and open-label tiotropium 18 μg. We measured physical activity during treatment with indacaterol 150 μg and matched placebo.

**Methods:**

We performed a randomized, three-period, cross-over study (21 days of treatment separated by two wash-out periods of 13 days) with indacaterol 150 μg or matching placebo and tiotropium 18 μg. Lung function was assessed by body plethysmography and spirometry. Physical activity was measured for one week by a multisensory armband at the end of both treatment periods with indacaterol/matched placebo. The primary endpoint was peak inspiratory capacity at the end of each treatment period.

**Results:**

129 patients (mean age, 61 years; mean post-bronchodilator FEV_1_, 64%), were randomized and 110 patients completed the study. Peak inspiratory capacity was 0.22 L greater with Indacaterol at day 21 compared to placebo (p < 0.001). Similar results were observed for tiotropium. Both bronchodilators also significantly improved other parameters of lung hyperinflation compared with placebo. All parameters of physical activity were significantly increased during treatment with indacaterol versus placebo.

**Conclusions:**

Indacaterol 150 μg improved lung hyperinflation in patients with moderate COPD, which was associated with an increase of physical activity.

**Trial registration:**

ClinicalTrials.gov registration number: NCT01012765.

## Background

Chronic obstructive pulmonary disease (COPD) is characterized by persistent airflow limitation that results in air trapping and lung hyperinflation [1–3]. Lung hyperinflation negatively affects various physiological responses to exercise and is thought to be one of the main mechanisms leading to exertional dyspnea, exercise intolerance, and, consequently, reduced physical activity in daily life [3, 4].

Significant limitations of physical activity are already present in patients with moderate COPD [5, 6]. This observation might be of clinical relevance as reduced physical activity is related to hospitalizations [7–9], impaired health-related quality of life [10], muscle deconditioning [10, 11], and all-cause mortality in patients with COPD [8, 12, 13].

Bronchodilators are central to symptomatic management of COPD [1]. Indacaterol is an inhaled ultra-long-acting β_2_-agonist providing 24-h bronchodilation with once-daily dosing in patients with COPD [14]. Indacaterol has been demonstrated to improve airflow limitation, dyspnea, and exercise intolerance in patients with COPD [14–17].

Less is known about the effects of treatment with indacaterol on lung hyperinflation and physical activity in patients with COPD. Rossi and colleagues showed that a single inhalation of indacaterol 150 μg reduced lung hyperinflation with treatment effects being slightly superior to the effects of a single inhalation of tiotropium 18 μg in patients with moderate COPD [18]. However, it is currently unknown whether these effects on lung function are sustained over a longer period of time. In another study, O’Donnell and colleagues evaluated the effects of indacaterol 300 μg on exercise endurance, lung hyperinflation, and physical activity in patients with moderate to severe COPD [17]. While significant improvements of exercise endurance time and hyperinflation during exercise could be observed, there was no effect on physical activity in that study [17]. This contrasts with a recent study demonstrating that open-label treatment with indacaterol resulted in an improvement of physical activity in 23 patients with COPD [19]. Applying a recently published measurement protocol with a rigorous whole-day measurement of physical activity [5] we decided to implement an accelerometer-based measurement of physical activity in the present study in order to evaluate whether an improvement of lung hyperinflation might also translate into changes of physical activity.

The present study was designed to assess the effects of indacaterol on lung hyperinflation compared with placebo and tiotropium, and to measure physical activity during treatment with indacaterol and matched placebo in patients with moderate COPD. Some study results have previously been reported in an abstract [20].

## Methods

### Patients

Patients eligible for inclusion in this study were male and female adults aged 40 years or older with a diagnosis of moderate COPD according to the spirometric classification of the “Global Initiative for Chronic Obstructive Pulmonary Disease” (post-bronchodilator forced expiratory volume in one second (FEV_1_) <80% and ≥50% of the predicted normal value and a post-bronchodilator ratio of FEV_1_ to forced vital capacity (FVC) <70%) and a smoking history of at least 10 pack years. Key exclusion criteria were a respiratory tract infection or exacerbation within 6 weeks prior to study entry, a history of asthma or any corticosteroid use (inhaled or systemic application) within the last three months prior to study entry, any other concomitant lung disease, or any clinically significant condition which in the opinion of investigator might compromise patient safety or compliance, interfere with evaluation, or preclude completion of the study.

### Study design

This was a randomized, multicenter, double-blind, placebo-controlled, 3-period, cross-over study conducted at 28 specialised respiratory care centres in Germany (ClinicalTrials.gov registration number: NCT01012765). The study protocol and all amendments were approved by the Ethics Committee of the University of Marburg (126/09A). The study was conducted according to the ethical principles of the Declaration of Helsinki between November 2009 and January 2011. All patients provided written informed consent prior to their participation in the study.

### Study medications

Following a 2-week run-in, patients were randomised to the treatment sequences with indacaterol 150 μg q.d. via single-dose dry powder inhaler (Onbrez® Breezhaler® inhalation powder; Novartis, Basel, Switzerland) or tiotropium 18 μg q.d. via its proprietary single-dose dry powder inhaler (Spiriva® HandiHaler® inhalation powder; Boehringer Ingelheim, Ingelheim, Germany) or placebo to indacaterol via single-dose dry powder inhaler (Breezhaler® device; Novartis, Basel, Switzerland). This means that treatment with indacaterol and placebo was double blinded, whereas treatment with tiotropium was open label. Each treatment period consisted of 21 days of dosing separated by 13 days of wash-out. Patients were randomized equally to one of six treatment sequences.

### Concomitant medications

Patients were provided with a short-acting β_2_-agonist (salbutamol) to use as required. Apart from study treatments, no other bronchodilator use was permitted. Long acting bronchodilators were discontinued prior to randomization with an appropriate washout of two days for long-acting β_2_-agonists and seven days for tiotropium and theophylline.

### Assessments

#### Lung function

Lung function was measured by body plethysmography and forced spirometry according to current recommendations [21, 22]. Three acceptable measurements had to be performed and the average values of functional residual capacity (FRC; intrathoracic gas volume [ITGV]) and inspiratory capacity (IC) were used for calculation of total lung capacity (TLC). Residual volume was calculated by subtracting the largest slow vital capacity assessed during expiration from TLC. Specific airway resistance (sRaw) was measured using the calculation of the effective specific airway resistance (sReff) [23]. Forced spirometry manoeuvres were performed after the body plethysmography measurements. The highest FEV_1_ and highest FVC of three acceptable manoeuvres were taken for analysis, irrespective whether they were derived from the same curve.

#### Physical activity

Physical activity was measured using the SenseWear multisensory armband (SenseWear® Armband®; BodyMedia, Pittsburgh, PA, USA) over a period of one week at the end of each treatment period as previously described [5]. Physical activity parameters were steps per day, minutes of at least moderate physical activity per day, and the average physical activity level (total daily energy expenditure divided by resting energy expenditure) as previously reported [5]. Validity of the energy-related physical activity estimates of the multisensory armband has previously been demonstrated [24, 25].

We defined the measurement of physical activity to be reliable when at least three days of measurement with a recorded wearing time of at least 22 hours per day were available, which has previously been shown to result in an intra-class correlation coefficient of about 0.7 in patients with moderate COPD [5]. An intra-class correlation coefficient of 0.7 indicates that about 70% of the variation of physical activity of the group is captured [5].

### Objectives

The primary objective of this study was to demonstrate the superiority of indacaterol 150 μg q.d. compared to placebo on peak IC after 21 days of treatment. The key secondary end-point was non-inferiority of indacaterol 150 μg q.d. compared to open label tiotropium 18 μg q.d. on peak IC after 21 days and trough IC after 20 days of treatment. Further secondary endpoints included the effects of indacaterol and tiotropium compared to placebo on trough and peak FRC, RV, RV/TLC ratio, sRaw, and FEV_1_. Peak values were defined as the individual highest value measured at 30, 120, 180 and 240 min post inhalation of study medication at day 21. Trough values were assessed 30 min prior inhalation of study drug at day 21. Exploratory endpoints were steps per day, minutes of at least moderate activity, and the physical activity level.

### Sample size and statistical methods

A treatment difference of 150 mL (with a standard deviation of 400 mL) in inspiratory capacity at day 21 was prespecified for non-inferiority. Based on this, a sample size of 80 evaluable patients was needed to detect this difference as statistically significant at the 2.5% level (one-sided) with 90% power. This sample size would provide 90% power for testing superiority, assuming a superiority margin of 300 mL (5% significance level, two sided). An assumed drop-out rate of 30% gave a minimum sample size of 125 patients.

Two patient populations were defined for analysis: 1) full-analysis-set population (n = 129), which comprised all randomized patients who received at least one dose of study drug during at least one study period; and 2) safety population (n = 129), which comprised patients who received at least one dose of study drug during at least one study period, and who had at least one safety assessment after baseline.

The analysis was performed comparing treatments with respect to the efficacy variables in an analysis of variance (ANOVA) model with the factors center, period, treatment and patient within center. Multiplicity issues were dealt with by *a priori* ordering of the hypotheses. Raw as well as adjusted least squares (LS) means were provided as point estimates for all pair-wise treatment contrasts. Non-inferiority of indacaterol to tiotropium was to be demonstrated if the 95% confidence interval for the mean inspiratory capacity difference of indacaterol minus tiotropium was entirely to the right of (higher than) 150 mL. The superiority of indacaterol vs. placebo was tested first within the confirmatory strategy for the full-analysis-set population. Once this superiority had been established, non-inferiority of indacaterol vs. tiotropium was analysed. Then, in the third step, superiority of indacaterol vs. tiotropium was tested. As soon as one of this hypothesis could not be established (i.e., the corresponding null-hypothesis failed to be rejected), all following hypotheses were interpreted as being purely exploratory and as not providing any confirmatory evidence. For all pair wise comparisons, the p-values from the primary ANOVA model were compared to those obtained by applying a non-parametric test (Wilcoxon signed rank) as supportive analysis. Secondary variables were analysed analogously to the primary endpoint.

## Results

The baseline demographics and clinical characteristics of all patients are given in Table 1. All patients were Caucasians (67.4% male) with a mean age of 61.4 years and a mean post-bronchodilator FEV_1_ of 64.0% predicted.

**Table 1:** **Demographics and baseline characteristics**

	Total N = 129
Age (years) Mean (SD)	61.4 (8.9)
Sex–Male, Female, %	67.4, 32.6
Duration of COPD (years), Mean (SD)	6.2 (6.1)
Ex-smoker/smoker, %	46.5/53.5
Pack-years in ex-smokers, mean (SD)	41.1 (16.4)
Pack-years in smokers, mean (SD)	41.0 (16.7)
FEV_1_ pre-bronchodilator, L, mean (SD)	1.71 (0.58)
FEV_1_ post-bronchodilator, L, mean (SD)	1.90 (0.51)
FEV_1_ reversibility, %	10.26
FEV_1_% predicted (post-bronchodilator), mean (SD)	64.02 (9.38)
FEV_1_/FVC post-bronchodilator, %	58.98 (10.29)

FEV1, forced expiratory volume in one second; FVC, forced vital capacity.

Of 129 patients screened, all were randomised and 110 (85.3%) completed the study. 19 patients discontinued due to adverse events (4 events), abnormal laboratory value (1 event), unsatisfactory therapeutic effect (3 events), protocol deviation (3 events), consent withdrawal (5 patients) and administrative problems (3 events). One patient discontinued the trial during the treatment period with indacaterol, 6 patients during the placebo period, and 3 patients during the treatment with tiotropium.

### Primary endpoint - peak IC

**Figure 1:**
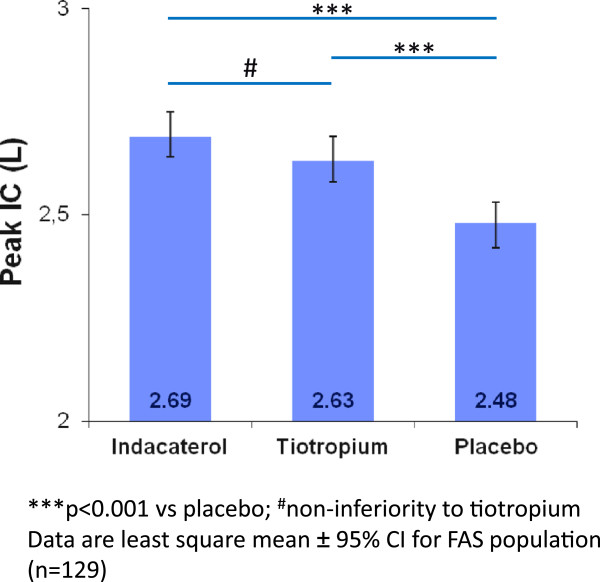
**Effect of indacaterol and tiotropium versus placebo on peak inspiratory capacity (IC) on Day 21.** ***p < 0.001 vs placebo; ^#^non-inferiority to tiotropium. Data are least square mean ± 95% CI for FAS population (n = 129).

Mean peak IC at Day 21 was 2.69 L and 2.48 L with indacaterol and placebo, respectively. The LSM treatment difference (220 mL, 95% CI 0.14–0.29) met the pre-defined criteria for superiority (p < 0.001) (Figure 1). The significant differences observed by the ANOVA model were confirmed by the results of the non-parametric Wilcoxon test (difference indacaterol vs. placebo, p < 0.001).

### Secondary endpoints - lung function

**Figure 2:**
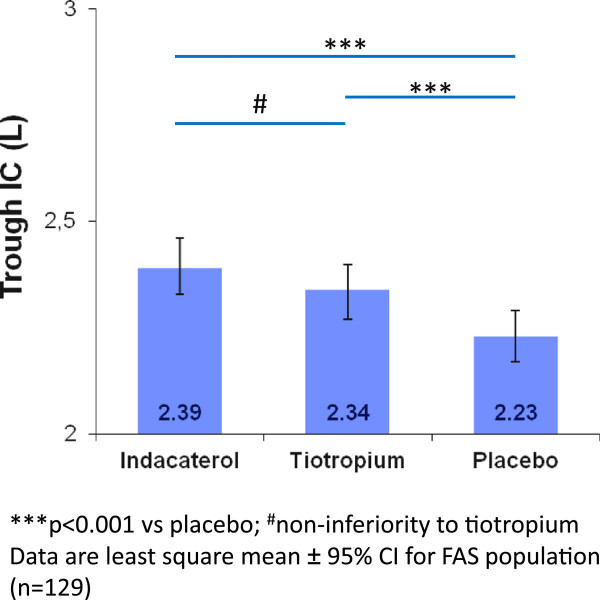
**Effect of indacaterol and tiotropium versus placebo in trough inspiratory capacity (IC) on Day 20.** ***p < 0.001 vs placebo; ^#^non-inferiority to tiotropium. Data are least square mean ± 95% CI for FAS population (n = 129).

Mean peak IC at Day 21 was 2.69 L with indacaterol and 2.63 L with tiotropium (open-label) with the LSM treatment difference (60 mL, 95% CI: -10–130) meeting the criteria for non-inferiority (p < 0.001). Mean trough IC at Day 20 was 2.39 L, 2.34 L and 2.23 L following treatment with indacaterol, tiotropium, and placebo, respectively (Figure 2). The results showed significant LSM treatment differences versus placebo favoring indacaterol (170 mL, 95% CI: 90–240; p < 0.001) and tiotropium (110 mL, 95% CI: 30–190; p = 0.005). Lung volumes and specific airway resistance demonstrated statistically significant treatments effects of both bronchodilators compared with placebo on both peak and trough values with no significant treatment differences between indacaterol and tiotropium (Table 2).

**Table 2:** **Effect of indacaterol and tiotropium versus placebo on lung function**

		IND vs PBO	TIO vs PBO
ITGV	Peak	-0.41 L p < 0.001; 95% CI: -0.54– -0.29	-0.38 L p < 0.001; 95% CI: -0.51– -0.25
	Trough	-0.27 L p < 0.001; 95% CI: -0.40– -0.14	-0.21 L p = 0.002; 95% CI: -0.35– -0.08
RV	Peak	-0.40 L p < 0.001; 95% CI: -0.54– -0.25	-0.39 L p < 0.001; 95% CI: -0.53– -0.25
	Trough	-0.32 L p = 0.001; 95% CI: -0.48– -0.15	-0.25 L p = 0.003; 95% CI: -0.42– -0.09
TLC	Peak	-0.16 L p = 0.021; 95% CI: -0.30– -0.03	-0.20 L p = 0.006; 95% CI: -0.34– -0.06
	Trough	-0.08 L p = 0.320; 95% CI: -0.23–0.07	-0.11 L p = 0.170; 95% CI: -0.26–0.05
IRV	Peak	0.12 L p = 0.003; 95% CI: 0.04–0.19	0.08 L p = 0.049; 95% CI: 0.00–0.15
	Trough	0.17 L p < 0.001; 95% CI: 0.09–0.25	0.11 L p = 0.005; 95% CI: 0.03–0.19
sRaw	Peak	-1.02 kPa*sec p < 0.001; 95% CI: -1.23– -0.81	-1.08 kPa*sec p < 0.001; 95% CI: -1.29– -0.87
	Trough	-0.81 kPa*sec p < 0.001; 95% CI: -1.00– -0.62	-0.63 kPa*sec p < 0.001; 95% CI: -0.83– -0.44
FEV_1_	Peak	0.24 L p < 0.001; 95% CI: 0.20–0.28	0.24 L p < 0.001; 95% CI: 0.19–0.28
	Trough	0.19 L p < 0.001; 95% CI: 0.15–0.24	0.17 L p = 0.001; 95% CI: 0.13–0.22

ITGV, intrathoracic gas volume; RV, residual volume; TLC, total lung capacity; IRV, inspiratory reserve volume; sRaw, specific airway resistance; FEV1, forced expiratory volume in one second.

Peak FEV_1_ at Day 21 was 2.00 L, 1.99 L and 1.76 L after indacaterol, tiotropium, and placebo treatment, respectively. The mean difference versus placebo was 240 mL for both indacaterol (95% CI: 200–280, p < 0.001) and tiotropium (95% CI: 190–280, p < 0.001). Trough FEV_1_ at Day 20 was 1.80 L, 1.78 L and 1.61 L after treatment with indacaterol, tiotropium and placebo, respectively (Table 2).

### Exploratory endpoints - physical activity

**Figure 3:**
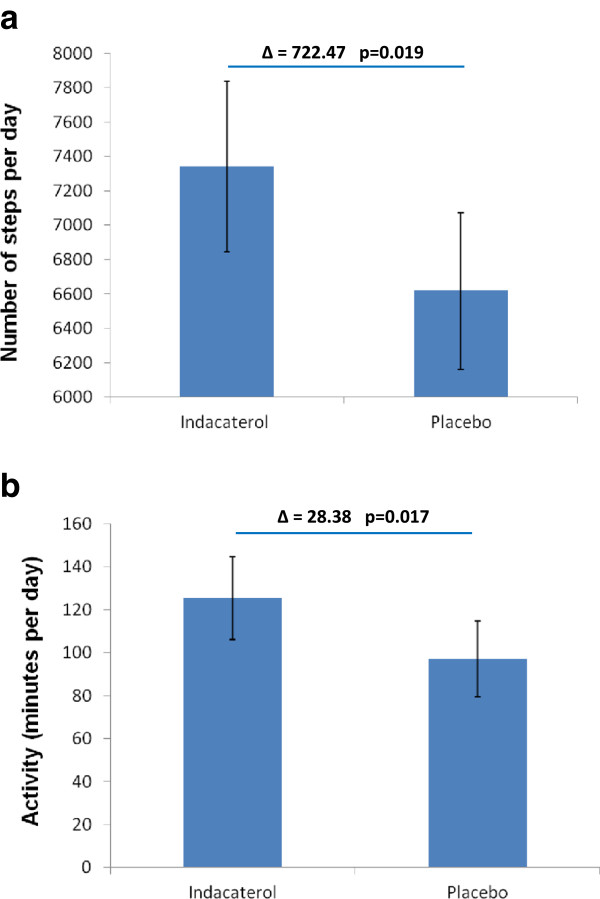
**Effect of indacaterol on physical activity during treatment. (a)** Steps per day (n=83) **(b)** Minutes of at least moderate physical activity (n=83).

Based on the prespecified reliability criteria with regard to wearing time of the accelerometer (at least three days of measurement with a recorded wearing time of at least 22 hours per day) complete datasets for the comparison of physical activity during treatment with indacaterol and placebo were available for 83 patients. Indacaterol significantly increased the total number of steps per day (7341; 95% CI: 6843–7838) compared with placebo (6618; 95% CI: 6162–7074) by 722 steps per day (p = 0.019; Figure 3a). Minutes of at least moderate activity per day were 125 min (95% CI: 106–145) during treatment with indacaterol versus 97 min (95% CI: 79–115) with placebo (p = 0.017; Figure 3b). Also the physical activity level was significantly higher during treatment with indacaterol (physical activity level, 1.61; 95% CI: 1.56–1.66) compared to placebo (physical activity level, 1.54; CI: 1.49–1.58) (p = 0.014). Further sensitivity analyses for the comparison of physical activity during treatment with indacaterol and placebo in 77 patients, who wear the accelerometer for at least 22 hours on at least 4 days, confirmed the significant differences between indacaterol and placebo (data not shown). A correlation between changes of IC and changes of any physical activity parameter could not be demonstrated (data not shown).

### Safety

The overall incidence of adverse events was generally low during treatment with indacaterol (24.6%), tiotropium (20.2%) and placebo (20.0%) (Table 3). Five out of the 129 patients (3.9%) experienced serious adverse events (one during treatment with indacaterol; one during treatment with placebo; three during treatment with tiotropium) not considered to be related to study treatment by the investigator. No death occurred in any of the groups.

**Table 3:** **Adverse events overall and most commonly occurring (≥2% of patients)**

	Indacaterol (N = 118) n(%)	Tiotropium (N = 119) n(%)	Placebo (N = 120) n(%)
Any adverse event (% of patients)	29(24.6)	24(20.2)	24(20.0)
Nasopharyngitis	9(7.6)	3(2.5)	8(6.7)
Back pain	2(1.7)	5(4.2)	2(1.7)
Headache	2(1.7)	2(1.7)	3(2.5)
Dyspnea	3(2.5)	1(0.8)	2(1.7)
Cough	4(3.4)	0(0.0)	0(0.0)
Rhinitis	2(1.7)	0(0.0)	0(0.0)
Hypotension	0(0.0)	0(0.0)	2(1.7)

## Discussion

The main findings of the present study are that indacaterol and tiotropium provided significant improvements in lung hyperinflation after three weeks of treatment, and that physical activity measured by a multisensory accelerometer was significantly improved during treatment with indacaterol compared to placebo.

Lung hyperinflation may be more closely associated with patient-reported symptoms like dyspnea and physical activity limitation than maximal expiratory flow rates such as FEV_1_ [2, 26]. The rationale behind the use of long-acting bronchodilators is the sustained reduction of lung hyperinflation with an increase of IC, which in turn is related to an improvement of exertional dyspnea and exercise intolerance in COPD [2, 26]. Unlike FEV1, a generally accepted minimal clinically important difference has not yet been identified for changes of IC following a therapeutic intervention, even though an increase of 100 mL can be considered to be potentially clinically meaningful [27]. The improvements of peak and trough IC with both bronchodilators observed in this study are in line with previously reported improvements of IC for indacaterol and tiotropium [18, 28, 29]. The increase of peak IC after 21 days of treatment with indacaterol 150 μg in the present study is slightly higher than the increase of peak IC by 177 mL reported by Rossi and colleagues after single administration of indacaterol 150 μg in moderate COPD [18] and the increase of IC by 170 mL 75 min after the administration of indacaterol 300 μg following three weeks of treatment observed in a recent study [28]. O’Donnell and colleagues observed an increase of peak IC and trough IC of 250 mL and 100 mL, respectively after 21 days of treatment with tiotropium 18 μg [28]. In another study Celli and colleagues observed even higher increases of both peak and trough IC following 4 weeks of treatment with tiotropium 18 μg [29].

Comparing the improvements of FRC and RV observed in our study with previously reported changes of both lung volumes after treatment with indacaterol or tiotropium it can generally be noted that the improvements fit with previous studies [18, 28, 29]. Reduction of FRC by about 300 mL and of RV by about 400 mL have been reported after single administration of indacaterol 150 μg before. For tiotropium the observed changes of FRC and RV in our study are in the range of previously reported improvements following 21 days treatment [28, 29].

While previous studies with tiotropium and indacaterol 300 μg demonstrated clear improvements of exercise endurance time measured through constant-load cycle ergometry testing [16, 17, 28] the effects of long-acting bronchodilation on physical activity in daily life are less studied so far, even though it is hypothesized that the improvements observed by this laboratory exercise test might also translate into an increase of physical activity in daily life [3]. However, measuring the physiological attributes that relate to the ability to perform physical activity might only indicate what a person is capable of doing, whereas a measurement of physical activity reflects what a person actually does, e.g. domestic work, occupational activities, and leisure-time activities [30, 31]. In the present study we were able to demonstrate that physical activity measured by a multisensory accelerometer improved during treatment with indacaterol compared with placebo. Steps per day and minutes of at least moderate activity per day significantly improved by 10.9% and 29.2%, respectively. The improvement of the physical activity level from 1.54 during placebo to 1.61 during treatment with indacaterol corresponded to an increase of energy expenditure related to physical activity by 12.9%. A minimal clinically important difference for changes of physical activity is not yet available for patients with COPD. However, our results can be discussed in the context of existing data with regard to mortality that is associated with reduced levels of physical activity. Waschki et al. demonstrated that the decrease of the physical activity level by 0.14 and the decrease of 1845 steps per day in a cohort of patients with mild to very severe COPD is associated with an increase of the relative risk of death by 117% and 104%, respectively [12]. Furthermore, recent data in healthy individuals suggest that 15 min of moderate-intensity exercise (e.g. brisk walking) a day is associated with 14% reduced risk of all-cause mortality [32].

The improvements of physical activity observed in our study confirm a recent open-label study with Indacaterol [19]. Using a different type of accelerometer Hataji and colleagues reported a significant increase of the number of steps per day by 26%, duration of at least moderate physical activity by 70%, and energy expenditure by 30% during four weeks of treatment [19]. However, open-label studies might be difficult to interpret in the context of such a novel outcome variable like physical activity, which might be highly influenced by behaviour and motivation [33]. This potential bias from an open label design brought us to the decision not to measure physical activity in our study during open-label treatment with tiotropium, while the lung function measurements might be less affected by open-label therapy with tiotropium in our study [34]. The effects of tiotropium on physical activity are subject to a different trial [35].

A recent double-blind study with indacaterol 300 μg in patients with moderate to severe COPD could not demonstrate any changes of objectively measured physical activity, even though significant improvements of exercise endurance time along with a decrease of IC could be observed [17]. It is difficult to interpret the contrasting observations of both studies. One explanation could be that we included patients with moderate COPD only, which might impact the magnitude of response. Another explanation for the significant effect on physical activity in our study might be the higher number of included patients or a more rigorous adjustment for wearing time. This, however, is clearly subject to speculation and further studies have to confirm our findings.

## Conclusion

To conclude this study demonstrated the benefit of indacaterol 150 μg on static lung hyperinflation, which was similar to the effects of tiotropium. This study also showed the beneficial effects of bronchodilator treatment with indacaterol on physical activity in daily life in patients with moderate COPD.
